# Supplementary material for: Enhancing the Properties of Liquid Crystal Polymers and Elastomers with Nano Magnetic Particles
Source: Materials (Basel). 2024 Oct 30;17(21):5273. doi: 10.3390/ma17215273 (PMC11547613; doi:10.3390/ma17215273)
Supplement: Supplementary file 1 [file materials-17-05273-s001.zip › materials-3203573-supplementary.pdf]

Small-angle X-ray Scattering Measurements of Monodomain Samples of LCP (CBZ6) and various fractions of nanomagnetic particles

1. Methods

Small-angle X ray scattering (SAXS) experiments were performed using the Synchrotron Facility at the Daresbury Laboratory, Warrington, and, in particular, Beam Line 16.1. An X-ray wavelength of 1.41 Å and a RAPID 2-D detector were used. The sample-to-detector distance was 3.12 m, providing a |Q| range of 0.01 $Å^{-1}$ to 0.14 $Å^{-1}$. The detector was calibrated using the collagen from a wet rat's tail; the SAXS pattern is a series of sharp orders occurring at regular intervals, for which the |Q| values are accurately known; this allows the pixels on the detector image to be converted to Q values [17].

2. Materials

Samples in the form of thin films on a Kapton substrate were prepared from CBZ6, a copolymer of I and II with 6mol% of II.

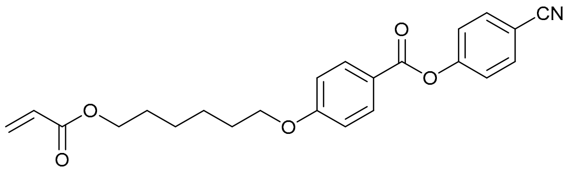

[I]

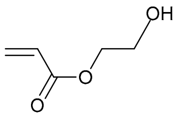

[II]

3. Results

Table S1 shows the composition of the films in terms of CBZ6 and 10 nm diameter spherical particles of magnetite.

Table S1
The composition of the samples used in the SAXS measurements.

Code	Wt % of Ferroparticles	$\langle P_2 \rangle$
C0	0.0	0.45
C1	0.07	0.47
C2	0.14	0.51

C3	0.28	0.50
C4	0.52	0.49
C5	1.04	0.44
C6	1.42	0.41
C7	2.84	0.25
C8	5.68	0.20

The samples were prepared on Kapton film and mounted on the beam line so that the incident X-ray beam was normal to the film surface and the direction of the previously applied magnetic field was vertical to the X-ray beam and vertical on the page in Figure S1.

Figure S1 shows the 2D small-angle scattering pattern of the monodomain C3, which shows strong scattering clustered around the zero-angle point. Although much of the scattering is obscured by the beam stop, it can be seen that it shows some highly anisotropic scattering which is very constrained in the vertical direction, indicating some highly extended objects, and it is spread out in the horizontal direction, showing that the extended objects are quite narrow in this direction. We believe such features to arise from individual chains. The strength of the scattering increases with an increasing volume fraction of the ferro particles and an increasing level of anisotropy, with increasing time in the magnetic field. A tentative analysis of these features by fitting a Gaussian peak to the observable data yields a width of the anisotropic object of 390 nm, with the length evaluation probably limited by the resolution of the setup but in excess of 5000 nm.

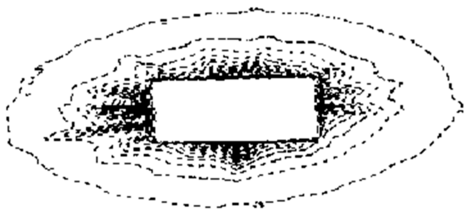

Figure S1: A contour plot of the 2D small-angle X-ray scattering pattern of the monodomain C3, containing CBZ6 plus 0.28% ferroparticles.

Clearly, the scattering shown in Figure S1 is the limit of the scattering geometry used in this work. It could be that it is better to use light scattering, and we performed some preliminary experiments of a 1% mixture of nanoparticles in kerosene. This provided evidence of highly extended objects of a length of 24 μm and a 5 μm width.

It proved difficult to perform reliable measurements of the mixtures of CBZ6 and nanoparticles. Despite the difficulties associated with Figure S1, we believe that this pattern provides strong evidence of the formation of highly anisotropic chains.

We also measured the scattering from the other monodomain samples listed in Table S1, focusing on the scattering measured at angles away from the beam stop. Lines were extracted horizontally (parallel to the field direction) and vertically (perpendicular to the field direction) across the image, producing plots of intensity against pixel. The pixel numbers were converted into q values using calibration graphs. Figure S2 shows a horizontal section of the 2D small-angle X-ray pattern of the monodomain shown in Figure S1 (CBZ6 plus 0.28% ferroparticles).

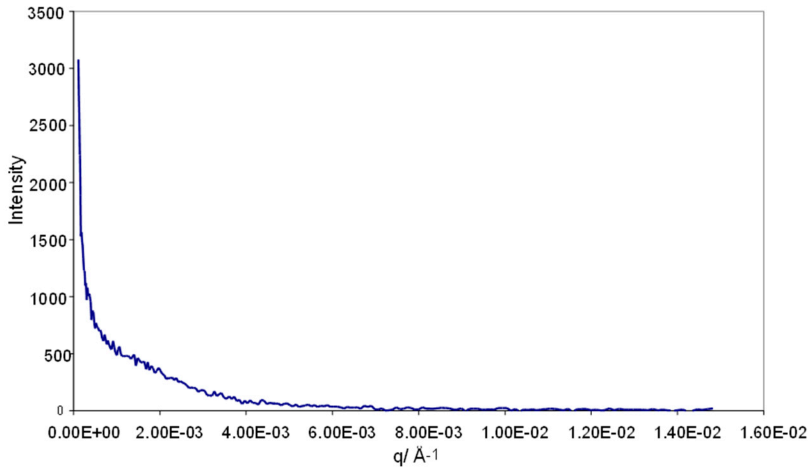

Figure S2: A horizontal section of the 2D small-angle X-ray pattern of the same monodomain (CBZ6 plus 0.28% ferroparticles) shown in Figure S1.

Figure S3 provides some of the first SAXS data for mixtures of liquid crystal polymers and ferro nanoparticles. It can be observed that initially the signal is good but towards the higher Q part the data becomes noisy due to a low signal. It is in this region that we would expect the characteristic oscillation of the particle form factor to be seen. If there is a degree of polydispersity in the size or indeed shape of the polymer, the averaging process could blur such oscillations out. As this is a relatively disordered system, it is possible to apply techniques developed from the understanding of fractal systems to the analysis of these curves. The scattering from such systems typically follow a power law behaviour, as exhibited by Equation S1, where I (q) is the scattering at a particular value of the scattering vector (q) ($q=4\pi\sin\theta/\lambda$, λ is the incident wavelength and 2θ is the scattering angle) and α and I_0 are constants. The value of α in particular provides information about the dimensionality of the material being studied [33]. To this end, plots of Log I(q) vs q were obtained for the samples described in Table S1, as shown in Figure S3.

$$I(q) = I_0 q^{-\alpha} \qquad \text{Equation S1}$$

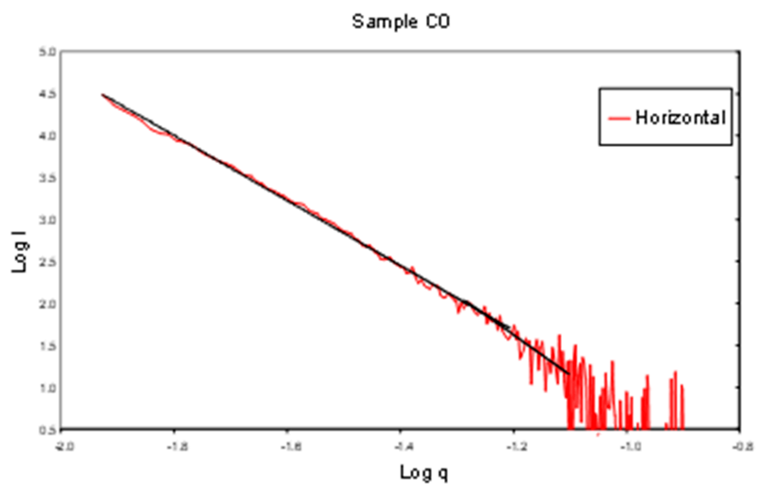

(a) Sample C0: 0% Ferroparticles

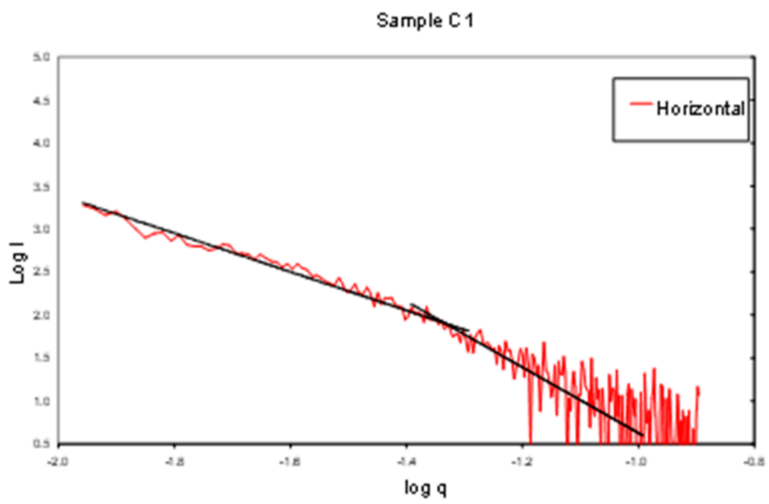

(b) Sample C1: 0.07% Ferroparticles

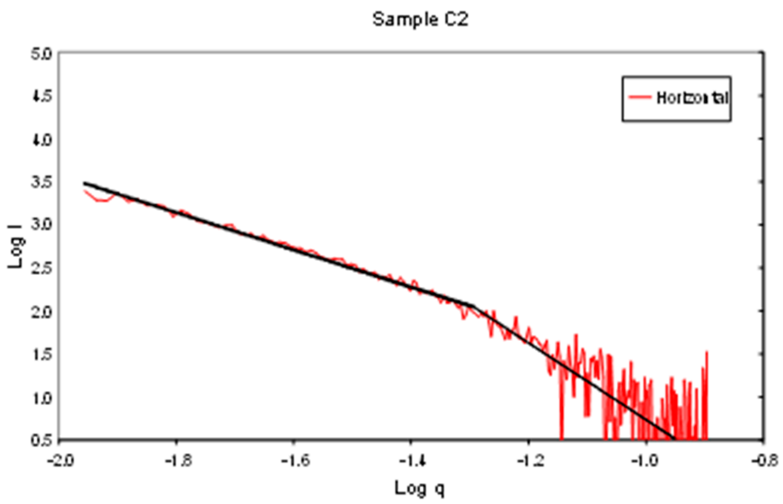

(c) Sample C2: 0.14% Ferroparticles

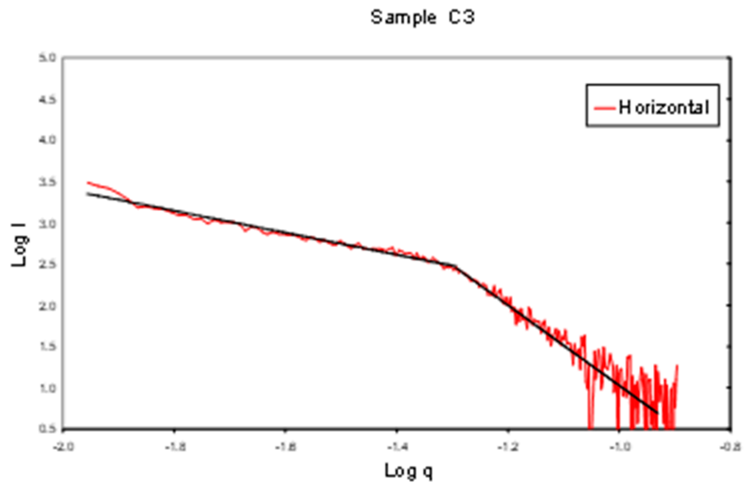

(d) Sample C3: 0.28% Ferroparticles

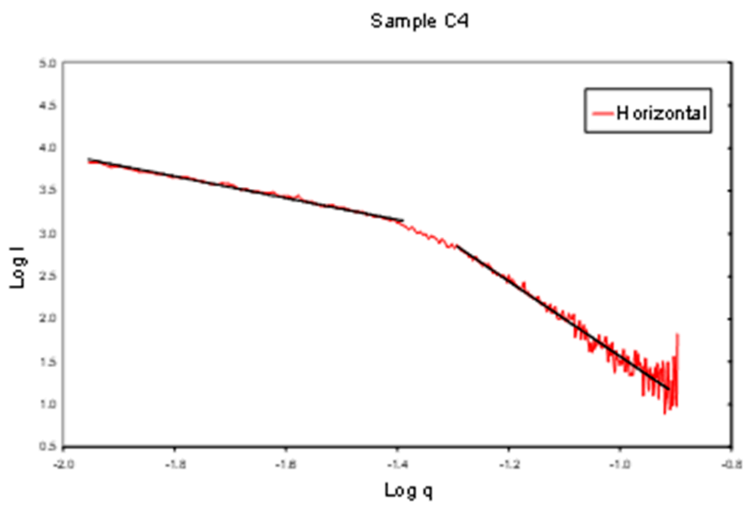

(e) Sample C4: 0.52% Ferroparticles

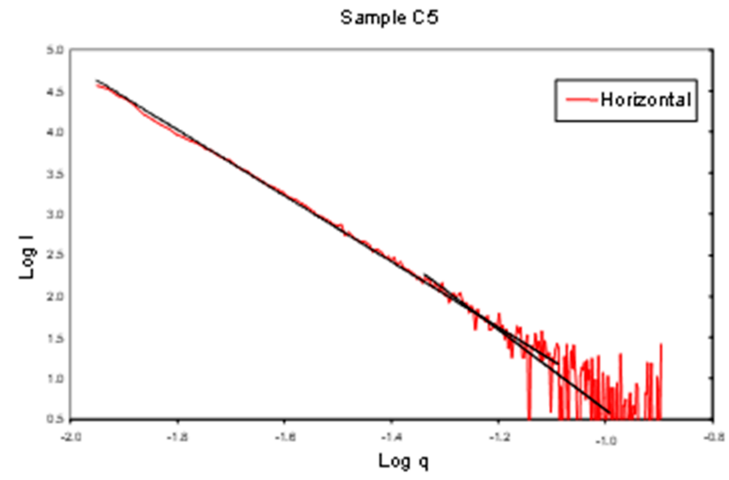

(f) Sample C5 1.04% Ferroparticles

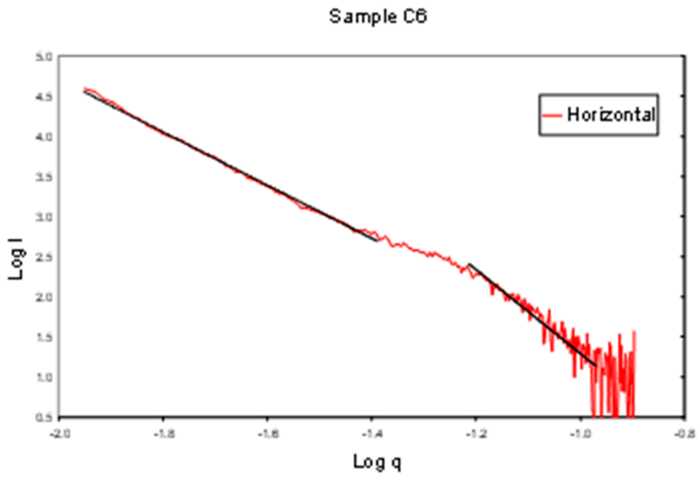

(g) Sample C6: 1.42% Ferroparticles

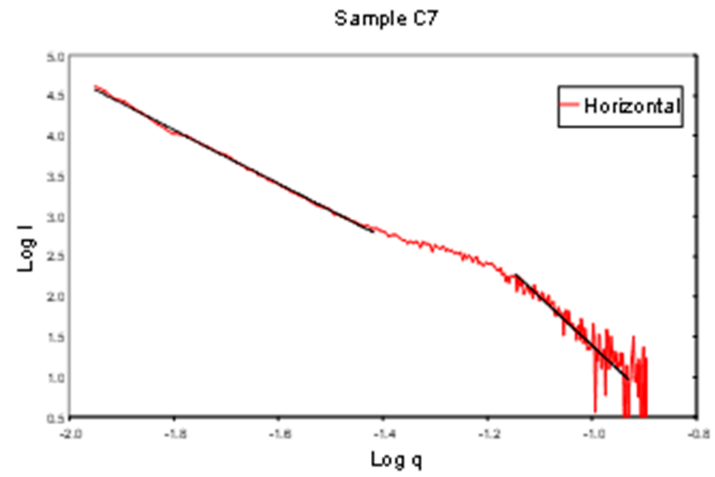

(h) Sample C7: 2.84% Ferroparticles

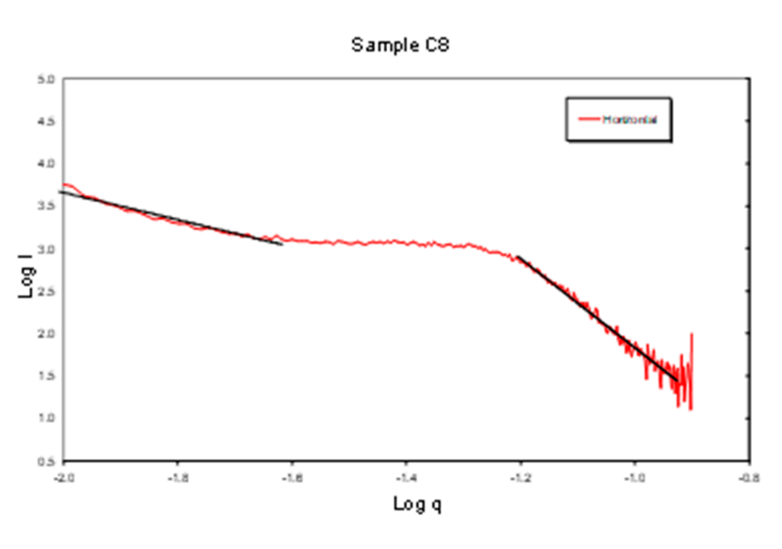

(i) Sample C8: 5.68% Ferroparticles

Figure S3: (a–i) Log–log plots used to determine the fractal numbers of the ferronematic polymer samples, with variations in the concentration of ferroparticles.

It has been found that plots of log I against Log Q provide a useful approach to investigating the possible aggregate structure of nanoparticles [34,35]. Figure S4 shows a plot of the exponent extracted from the slopes in the low Q portions of the scattering curves shown in Figure S3(a)–(i) for the different concentrations of nanoparticles. The slope gives the fractal number d, which would be equal to 1 for a rod-shaped structure and a larger number for a more space-filling structure such as a sphere. Examining the series of plots, a systematic variation can be observed, moving from a single straight line with no nanoparticles to plots with two straight sections of different slopes. At higher concentrations, a similar system is seen, but with an intermediate section. At the lowest concentrations, the fractal number drops from 2 to 1, indicating a 1D aggregate, but then at higher concentrations of 1% or above, the fractal number jumps to 4 and then reduces with increasing concentration. The lowest concentrations are evidence of an increasingly 1D structure, which at higher concentrations becomes a more space-filling aggregate, perhaps through the formation of a branched network. Clearly, when the nanoparticles form chains in an environment such as kerosene, the environment offers little constraint to the characteristics of the chaining. In a liquid crystal polymer environment, the situation is more complex due to the highly viscous polymer matrix and the presence of a liquid crystal phase that itself interacts with the magnetic field.

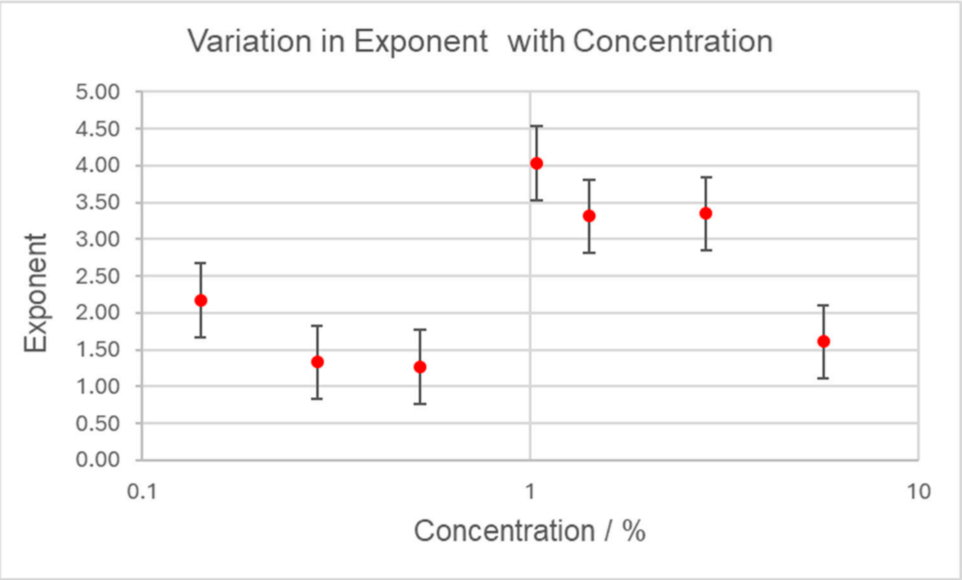

Figure S4: Fractal exponent variations, obtained from Figure S3, with concentration of ferroparticles.

4. Summary

In this Supplementary Material, we have described some of the small-angle X-ray scattering measurements taken of the monodomain samples prepared with varying amounts of ferroparticles. The SAXS data provide some evidence that supports the model of the formation of chains at low concentrations but, as the optical microscopy shows, at higher concentrations aggregation takes place which the SAXS data suggest is more 3D in nature. Clearly, this area requires a more comprehensive study to develop a thorough understanding of the aggregation process in liquid crystal environments.

References
